# The Impact of Digital Inequities on Nasal and Paranasal-Sinus Cancer Disparities in the United States: A Cohort Study

**DOI:** 10.2196/52627

**Published:** 2025-07-15

**Authors:** David J Fei-Zhang, Amelia Sherron Lawrence, Daniel C Chelius, Anthony M Sheyn, Jeffrey C Rastatter

**Affiliations:** 1 School of Medicine, Northwestern University Feinberg, 420 E Superior S, Chicago, IL, 60611, United States; 2 Elson S. Floyd College of Medicine, Washington State University, Spokane, WA, United States; 3 Department of Otolaryngology-Head and Neck Surgery, Baylor College of Medicine, Houston, TX, United States; 4 Department of Otolaryngology-Head and Neck Surgery, University of Tennessee Health Science Center, Memphis, TN, United States; 5 Division of Pediatric Otolaryngology, Ann & Robert H. Lurie Children’s Hospital of Chicago, Chicago, IL, United States; 6 Department of Otolaryngology-Head and Neck Surgery, Northwestern University Feinberg School of Medicine, Chicago, IL, United States

**Keywords:** paranasal sinus diseases, nasopharyngeal carcinoma, statistics, digital inequities, cancer disparities, technology, morality, treatment, care access, United States, cohort study, sinus cancer, sociodemographic, online access, equity, digital divide, public health

## Abstract

**Background:**

In the modern era, the use of technology can substantially impact care access. Despite the extent of its influence on several chronic medical conditions related to the heart, lungs, and others, the relationship between one’s access to digital resources and oncologic conditions has been seldom investigated in select pathologies among gastrointestinal and head-neck regions. However, studies on the influence of this “digital inequity” on other cancers pertaining to nasal and paranasal sinus cancer (NPSC) have yet to be performed. This remains in stark contrast to the extent of large data approaches assessing the impact of traditional social determinants/drivers of health (SDoH), such as factors related to one’s socioeconomic status, minoritized race or ethnicity, and housing-transportation status, on prognostic and treatment outcomes.

**Objective:**

This study aims to use the Digital Inequity Index (DII), a novel, comprehensive tool that quantifies digital resource access on an area- or community-based level, to assess the relationship between inequities in digital accessibility with NPSC disparities in prognosis and care in the United States.

**Methods:**

Patients with NPSC from 2008 to 2017 in the Surveillance, Epidemiology, and End Results Program were assessed for significant regression trends in the long-term follow-up period and treatment receipt across NPSCs with increasing overall digital inequity, as measured by DII. DII was based on 17 census-tract level variables derived from the summarized values overlapping that same time period from the US Census/American Community Survey and Federal Communications Commission Annual Broadband Report. Variables were categorized as infrastructure-access (ie, electronic device ownership, internet provider availability, and income-broadband subscription ratio) or sociodemographic (education, income, age, and disability), ranked, and then averaged into a composite score to encompass direct and indirect factors related to digital inequity.

**Results:**

Across 8012 adult patients with NPSC, males (n=5416, 67.6%) and White race (n=4293, 53.6%) were the most represented demographics. With increasing digital inequity, as measured by increasing total DII scores, significant decreases in the length of long-term follow-up were observed with nasopharyngeal (*P*<.01) and maxillary sinus cancers (*P*=.02), with decreases as high as 19% (35.2 to 28.5 months, nasopharynx). Electronic device and service availability inequities showcased higher-magnitude contributions to observed associated regression trends, while the income-broadband ratio contributed less. Significantly decreased odds of receiving indicated surgery (lowest odds ratio 0.87, 95% CI 0.80-0.95, maxillary) and radiation (lowest odds ratio 0.78, 95% CI 0.63-0.95, ethmoid) for several NPSCs were also observed.

**Conclusions:**

Digital inequities are associated with detrimental NPSC care and surveillance trends in the United States, even when accounting for traditional SDoH factors. These results prompt the need to include digital factors into the discussion of contextualizing SDoH-based analyses of cancer care disparities, as well as the specific factors from which prospective implementations and initiatives can invest limited public health resources to alleviate the most pertinent drivers of disparities.

## Introduction

Despite multiple studies highlighting the clinical factors of nasal and paranasal sinus cancer (NPSC) prognosis [1-4], the rare occurrence of NPSC has limited investigations of how nonclinical factors, namely social determinants/drivers of health (SDoH), come to influence prognosis. Among the few that have analyzed SDoH, most investigations have focused on components of socioeconomic status (SES) and race-ethnicity for their significant impactful associations with NPSC outcomes. For instance, Sharma et al [5] showed that patients with paranasal cancers had higher mortality and advanced staging on preliminary diagnosis associated with worse SES. In addition, London et al [6] concluded that SES alongside race-ethnicity significantly impacted survival, stage at diagnosis, and treatment in patients with nasopharyngeal carcinoma.

As the United States was plunged into the COVID-19 era, technology and internet access became crucial; yet, confounding elements in how SDoH affect clinical intervention use. During this period, telehealth modalities saw increasing importance in bringing patients with cancer from varying levels of SES equivalent means of health care, including through diagnostic testing and prescribing symptomatic treatment [5,7,8]. As this use became more prevalent, disparities in technology access became more apparent with a demonstrated potential to impact the already present associations between possessing worse social determinant factors and poor health outcomes [9].

This intersection between health equity and digitization of health care has brought a unique aspect of otolaryngologic care for vulnerable patient populations to the forefront. Among the few efforts to assess this relationship, Darrat et al [10] observed 1162 patients with head-neck malignancies from a single tertiary care center who showed worsening SES being associated with decreased use of telehealth. Despite this demonstrated impact on the local level, investigations of the health impact of these digital factors on a national scale have sparsely been performed, let alone for their effects on influencing NPSC care and prognosis.

Among the few initiatives to comprehensively assess this “digital inequity,” state-level efforts, such as the Digital Divide Index from the Rural Indiana Stats database, or national-level ones, such as the Federal Communications Commission Connect2Health Broadband Map, have been created. However, they have key flaws in not contextualizing digital access alongside traditionally investigated SDoH (ie, SES, race-ethnicity, and disability status), encompassing a limited geographic scope, or using old raw data sources [11,12].

To address this crucial deficit, this study aims to apply the Digital Inequity Index (DII) to assess how digital inequity is associated with NPSC disparities in the United States. This study hypothesized that increasing digital inequity would be associated with poorer NPSC outcomes in long-term follow-up and decreased receipt of indicated treatment modalities (ie, surgery and radiation therapy) while adjusting for validated, traditional SDoH.

**Figure 1.**
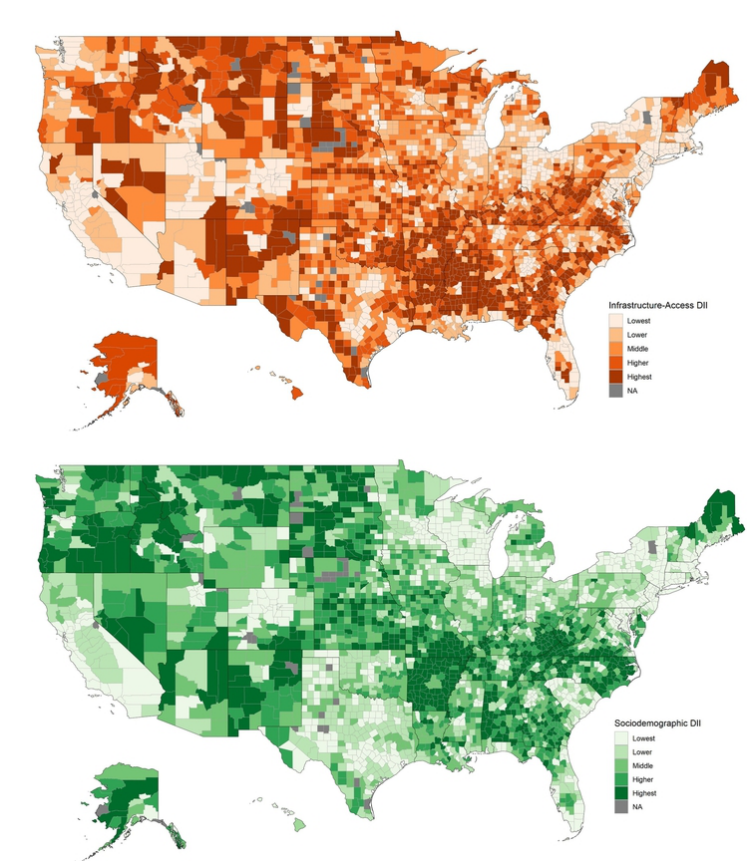
Distribution of total DII ranked scores across the United States. Ranked digital inequity scores in infrastructure access-usage and sociodemographic categories were assigned per county. DII: Digital Inequity Index.

The DII is a US-based, geographically differentiated tool that comprehensively assesses digital inequity across a variety of factors encompassing broadband infrastructure, electronic device access, and internet access affordability while adjusting for traditional SDoH nationally (Figure 1). It was previously developed and implemented as a means of using validated multivariate models of digital resources and traditional SDoH measures while sourcing from updated, publicly available data from the American Community Survey (ACS) and the US Census. Among prior uses of this tool, GI-aerodigestive cancers were observed to have vast differences in mortality, lack of follow-up care access, and disparities in first-line treatment receipt independent of the effects of traditional SDoH [13]. However, to our knowledge, there is a lack of investigations into whether digital inequities are associated with the care and prognosis of NPSC across a national patient population.

## Methods

### Overview

This research was conducted in Chicago, Illinois, United States, during the period of September 2022 to July 2023. No generative artificial intelligence was used in conducting any part of this research, nor any written parts of this manuscript.

### Ethical Considerations

Prior institutional review board/ethics committee approval or waiver of informed consent was exempted per Northwestern University’s institutional review board. The research is not considered participant research due to the databases queried consisting of publicly available, deidentified data and secondary analyses of such without additional institutional review board approval per the university.

### The DII

The DII was based on 17 census-tract level variables derived from the ACS 5-year estimates spanning 2008‐2017 and the Federal Communications 14th Broadband Report. Per ACS-derived methodologies, the use of the 5-year estimates allowed adjustments for differing chronological periods covered in order to adjust for temporal changes in digital access during this period. Variables were extracted and grouped into themes of “infrastructure-access” comprised of the measures representing “households without a desktop or laptop,” “without access to non-mobile broadband,” “without access to broadband: DSL,” “without access to broadband: cable,” “without access to broadband: fiber,” “without access to broadband: terrestrial fixed wireless,” “without a mobile or non-mobile internet subscription of any type,” “without an internet subscription of cable, fiber, or DSL,” “without a broadband subscription in households making $20,000 or less,” “without a broadband subscription in households making $20,000 - $74,999,” and “without a broadband subscription in households making $75,000 or more”; and “sociodemographic” comprised of “25+ aged people without high school diploma,” “25+ aged people without an associate’s degree or higher,” “25+ aged people without a bachelor’s degree or higher,” “below poverty level within the last 12 months,” “below 150% of poverty level within the last 12 months,” and “disability status pertaining to cognitive, ambulatory, or self-care difficulties.” Additionally, “infrastructure-access” variables were split into the subthemes of “Device Access,” “Internet Availability,” and “Income-Access.” These subthemes and variables have been summarized in Multimedia Appendix 1.

Ranked scores were then assigned to each ACS variable based on their relative value compared with all other census tracts nationwide. These were then adjusted by tract population to calculate weighted mean scores on the county level within their respective DII categories. DII scores were then arranged into 5 ordered classes by natural break (Jenks) classification by comparing the sum of squared deviations between classes to each array mean and using a goodness of variance fit. These 5 classes were then labeled as “Lowest,” “Lower,” “Middle,” “Higher,” and “Highest” (Figure 1). DII scores were abstracted and matched to patient data based on county of residence at the time of diagnosis.

### Patient Database and Clinicodemographic Variables

The National Cancer Institute-Surveillance, Epidemiology, and End Results Program (NCI-SEER) database contains national patient data on pathological characteristics, treatment modalities, and prognostic outcomes. It is the main program of the NCI in order to support cancer surveillance activities and acts as the national governmental authority for collecting information on cancer incidence and survival in the United States. These patient data were collected across 18 NCI-designated cancer registries across institutions from all regions of the United States, representing over 48% of the US population. Months under surveillance represent a length-of-care measurement reflecting the active follow-up a patient receives for their primary malignancy up until the last provider interaction. The end point was designated by the SEER database as the last point when a patient received care due to being lost to follow-up or experienced a mortal outcome per the SEER-designated variable of “vital status.” Delineations of months were the lowest-level strata available due to compliance with HIPAA (Health Insurance Portability and Accountability Act), deidentified status. Primary surgery and radiation occurrence represent whether patients received surgery or radiation for their primary malignancy.

### Population Definitions

SEER was queried for adult (20 years and older) patients diagnosed with NPSC from 2008 to 2017. Primary sites were extracted using the *ICD-O-3* (*International Classification of Diseases for Oncology, Third Edition*) topographic codes (C11.0‐11.9; C30.0‐31.9). Patients with incomplete data across the clinicodemographic variables were excluded from respective analyses necessitating those variates.

### Statistical Methods: Demographics Tables, Linear and Logistic Regressions, and Boxplots

Demographics tables were grouped by DII scores delineated by a natural break (Jenks) classifications of “Lowest,” “Lower,” “Middle,” “Higher” and “Highest.”

Months under surveillance within each primary malignancy were analyzed by DII-category scores. DII scores were split into relative, equivalently sampled quintiles based on actual DII scores within each primary malignancy. The relative-DII quintiles were delineated by “<20”, “20‐39.99,” “40‐59.99,” “60‐79.99,” and “80‐99.99” representing their relative percentiles per malignancy type (eg within disease A, patients with the lowest DII scores are grouped into the “<20” quintile group).

Among these DII-quintiles, differences between the mean months under the surveillance period for the lowest and highest DII-scored quintiles were calculated. Trend significance was assessed by linear regression across relative-DII quintiles for both continuous measures, and boxplots were generated to assess the median, IQR, and 1.5 times the IQR. Mean values were also calculated per relative quintile group.

Primary surgery and radiation occurrence within different malignancy types were analyzed with univariate logistic regression across relative-DII quintiles per DII category.

Statistical significance was set as *P* value <.05. Two-sided *P* values were reported for analyses. Analyses were conducted in R (version 4.2.3; R Project for Statistical Computing).

## Results

### Overview

A total of 8012 adult patients with primary NPSC were extracted from SEER including nasal cavity (n=1821, 22.7%), nasopharynx (n=4225, 52.7%), sinus ethmoid (n=276, 3.4%), sinus maxillary (n=1187, 14.8%), and sinus other (n=383, 4.8%). DII scores ranged from “Lowest” (n=6099, 76%) to “Highest” (n=172, 2.1%), signifying “lowest digital inequity” and “highest digital inequity,” respectively. Males (n=5416, 67.6%) and White race (n=4293, 53.6%) were the most represented in the study population. Further demographic and clinical characteristics stratified by DII are noted in Table 1.

**Table 1.** Patient characteristics by Digital Inequity Index (DII) score. DII categories represent increasing levels of digital inequity averaged across infrastructure access-usage and sociodemographic-related factors that comprise access to digital resources. “Lowest” to “Highest” represent classes divided by Jenks classification metrics.

Characteristics	DII category, n (%)				
	Lowest DII, N=6099 (76%)	Lower DII, N=1157 (14%)	Middle DII, N=365 (4.6%)	Higher DII, N=219 (2.7%)	Highest DII, N=172 (2.1%)
Age (years; N=8012), n (%)					
20‐44	938 (15.0)	146 (13.0)	44 (12.0)	24 (11.0)	23 (13.0)
45‐64	2820 (46.0)	554 (48.0)	168 (46.0)	93 (42.0)	74 (43.0)
65‐84	2015 (33.0)	411 (36.0)	140 (38.0)	90 (41.0)	67 (39.0)
85+	326 (5.3)	46 (4.0)	13 (3.6)	12 (5.5)	8 (4.7)
Sex (N=8012), n (%)					
Male	4111 (67.0)	792 (68.0)	258 (71.0)	143 (65.0)	112 (65.0)
Female	1988 (33.0)	365 (32.0)	107 (29.0)	76 (35.0)	60 (35.0)
Race (N=8012), n (%)					
White	2996 (49.0)	731 (63.0)	271 (74.0)	166 (76.0)	129 (75.0)
Asian or Pacific Islander	1843 (30.0)	110 (9.5)	21 (5.8)	0 (0.0)	0 (0.0)
Black	512 (8.4)	203 (18.0)	54 (15.0)	37 (17.0)	31 (18.0)
Hispanic	653 (11.0)	92 (8.0)	17 (4.7)	9 (4.1)	4 (2.3)
Unknown	69 (1.1)	15 (1.3)	2 (0.5)	0 (0.0)	1 (0.6)
Native American	26 (0.4)	6 (0.5)	0 (0.0)	7 (3.2)	7 (4.1)
Region (N=8012), n (%)					
Midwest	256 (4.2)	274 (24.0)	63 (17.0)	15 (6.8)	1 (0.6)
Northeast	961 (16.0)	120 (10.0)	0 (0.0)	0 (0.0)	0 (0.0)
South	779 (13.0)	440 (38.0)	255 (70.0)	187 (85.0)	149 (87.0)
West	4103 (67.0)	323 (28.0)	47 (13.0)	17 (7.8)	22 (13.0)
Primary site category (N=8012), n (%)					
Middle ear	95 (1.6)	17 (1.5)	5 (1.4)	2 (0.9)	1 (0.6)
Nasal cavity	1313 (22.0)	282 (24.0)	123 (34.0)	58 (26.0)	45 (26.0)
Nasopharynx	3335 (55.0)	561 (48.0)	152 (42.0)	96 (44.0)	81 (47.0)
Sinus ethmoid	199 (3.3)	52 (4.5)	12 (3.3)	7 (3.2)	6 (3.5)
Sinus maxillary	861 (14.0)	193 (17.0)	60 (16.0)	43 (20.0)	30 (17.0)
Sinus other	296 (4.9)	52 (4.5)	13 (3.6)	13 (5.9)	9 (5.2)
ICD-O-3^a^ histopathology (N=8012), n (%)					
Acinar cell neoplasms	4 (<0.1)	0 (0.0)	2 (0.5)	0 (0.0)	0 (0.0)
Adenomas and adenocarcinomas	562 (9.2)	110 (9.5)	38 (10.0)	27 (12.0)	19 (11.0)
Complex epithelial neoplasms	41 (0.7)	10 (0.9)	4 (1.1)	0 (0.0)	0 (0.0)
Complex mixed and stromal neoplasms	9 (0.1)	0 (0.0)	1 (0.3)	1 (0.5)	0 (0.0)
Cystic, mucinous, and serous neoplasms	10 (0.2)	6 (0.5)	2 (0.5)	1 (0.5)	0 (0.0)
Ductal and lobular neoplasms	14 (0.2)	3 (0.3)	1 (0.3)	1 (0.5)	0 (0.0)
Epithelial neoplasms (not otherwise specified)	1432 (23.0)	192 (17.0)	40 (11.0)	29 (13.0)	27 (16.0)
Mucoepidermoid neoplasms	58 (1.0)	8 (0.7)	6 (1.6)	1 (0.5)	0 (0.0)
Squamous cell neoplasms	3949 (65.0)	820 (71.0)	270 (74.0)	158 (72.0)	126 (73.0)
Transitional cell papillomas and carcinomas	20 (0.3)	8 (0.7)	1 (0.3)	1 (0.5)	0 (0.0)
TNM combined staging (N=7577), n (%)					
Stage I-III	3264 (57.0)	614 (55.0)	195 (56.0)	119 (57.0)	96 (58.0)
Stage IV and above	2478 (43.0)	496 (45.0)	154 (44.0)	91 (43.0)	70 (42.0)
Primary surgery performed (N=7904), n (%)					
No surgery	3790 (63.0)	691 (61.0)	192 (54.0)	128 (60.0)	104 (63.0)
Surgery	2238 (37.0)	451 (39.0)	163 (46.0)	85 (40.0)	62 (37.0)
Radiation therapy performed (N=8012), n (%)					
No therapy	1768 (29.0)	371 (32.0)	129 (35.0)	89 (41.0)	62 (36.0)
Therapy	4331 (71.0)	786 (68.0)	236 (65.0)	130 (59.0)	110 (64.0)
Vital status on last follow-up (N=8012), n (%)					
Alive	3832 (63.0)	668 (58.0)	212 (58.0)	112 (51.0)	98 (57.0)
Dead	2267 (37.0)	489 (42.0)	153 (42.0)	107 (49.0)	74 (43.0)

a *ICD-O-3*: *International Classification of Diseases for Oncology, Third Edition*.

### Malignancy-Type Trends in Months Under Surveillance by Relative DII Percentile

**Figure 2.**
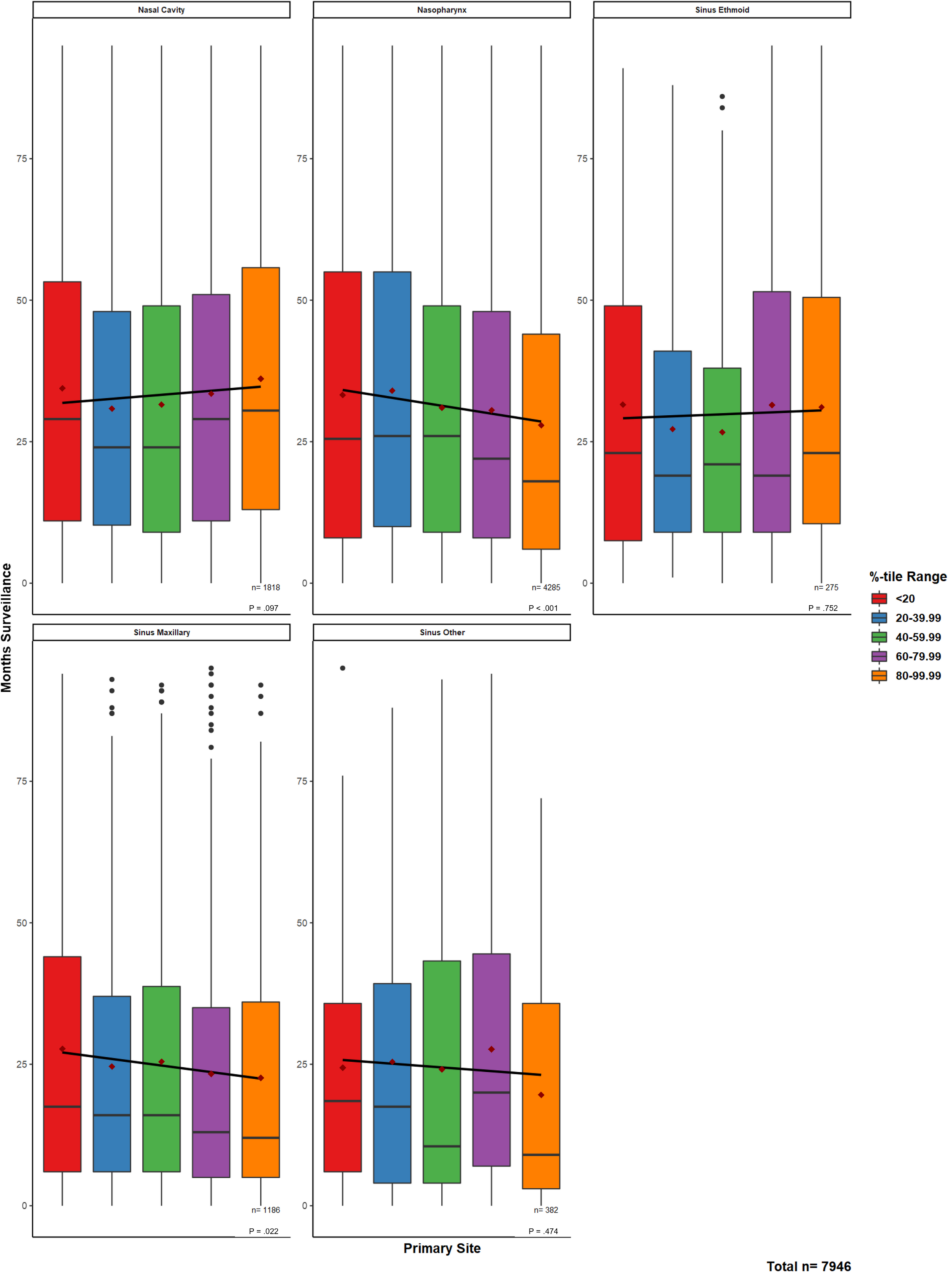
Linear regression trends in months surveyed across increasing Digital Inequity Index quintiles. Linear regressions across all the represented values (ie, not the mean values) in each of the boxplot quintiles were performed to assess for continuous trend significance of the surveillance period for increasing the total Digital Inequity Index. Boxplots=median, IQR, 1.5*IQR; mean months surveyed per quintile=maroon diamonds; outliers=blackdots; *P* value for regression.

**Figure 3.**
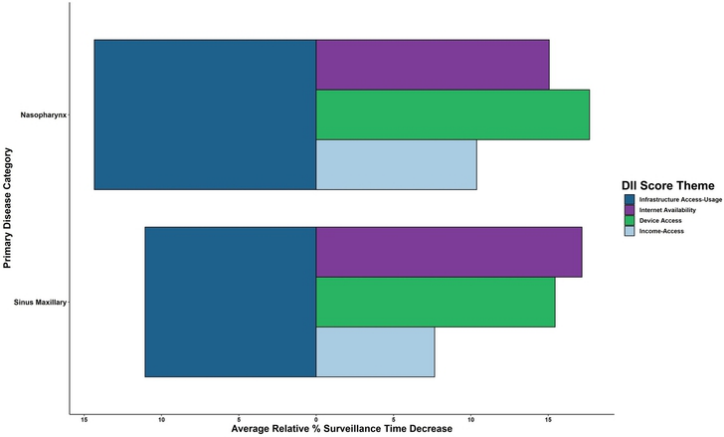
Relative decreases in mean months of surveillance with increasing DII scores. Percentage decreases from lowest to highest-DII quintiles based on mean months surveyed for DII-theme subscores. Patients with nasal and paranasal sinus cancer were assigned DII scores and split into relative quintiles. DII: Digital Inequity Index.

Substantial decreases in mean surveillance period were observed among patients with NPSC with the lowest (ie, having the least digital inequity) to the highest-DII quintiles. These decreases were significant and ranged from the following: 14.4% decreases in mean surveillance/follow-up period from 32.54 (SD 28) months to 27.85 (SD 26) months for nasopharyngeal cancers (*P*<.01), and 11.1% decreases from 26.37 (SD 26) months to 23.45 (SD 23) months in maxillary sinus cancers (*P*=.02; Figures2  3). Contributing to this overall trend, inequities in electronic device and service availability largely contributed to these decreases, while the income-broadband ratio contributed less (Figure 3).

### Malignancy-Type Trends in Surgery and Radiation Therapy by Relative DII

With increasing digital inequity/DII scores, patients with maxillary sinus showed markedly decreased odds of receiving indicated surgery for their primary tumor (odds ratio [OR] 0.87, 95% CI 0.80‐0.95; *P*=.01; Table 2). Similarly, increasing digital inequity/DII scores were associated with markedly decreased odds of receiving indicated radiation therapy for patients with primary ethmoid sinus (OR 0.78, 95% CI 0.63‐0.95; *P*=.01) and nasopharyngeal tumors (OR 0.90, 95% CI 0.85‐0.95; *P*<.01; Table 3).

**Table 2.** Increasing Digital Inequity Index trends with surgery receipt.

Primary site and characteristics		OR^a^	95% CI	*P* value
Nasopharynx				
Infrastructure access-usage		1.08	0.95-1.22	.22
Sociodemographic		1.11	0.99-1.26	.08
Nasal cavity				
Infrastructure access-usage		1.02	0.95-1.10	.57
Sociodemographic		1.03	0.95-1.11	.49
Sinus ethmoid				
Infrastructure access-usage		1.04	0.86-1.25	.68
Sociodemographic		1.08	0.90-1.30	.42
Sinus maxillary				
Infrastructure access-usage		0.87	0.80-0.95	.01
Sociodemographic		0.85	0.78-0.93	<.01
Sinus other				
Infrastructure access-usage		0.92	0.79-1.06	.25
Sociodemographic		0.89	0.77-1.03	.12

a OR: odds ratio.

**Table 3.** Increasing Digital Inequity Index trends with radiation receipt.

Primary site and characteristic	OR^a^	95% CI	*P* value
Nasopharynx			
Infrastructure access-usage	0.90	0.85-0.95	<.001
Sociodemographic	0.90	0.85-0.95	<.001
Nasal cavity			
Infrastructure access-usage	0.96	0.90-1.02	.21
Sociodemographic	0.99	0.93-1.06	.83
Sinus ethmoid			
Infrastructure access-usage	0.78	0.63-0.95	.01
Sociodemographic	0.80	0.65-0.98	.03
Sinus maxillary			
Infrastructure access-usage	0.94	0.86-1.02	.16
Sociodemographic	0.98	0.90-1.06	.61
Sinus other			
Infrastructure access-usage	0.90	0.77-1.04	.15
Sociodemographic	0.89	0.77-1.03	.13

a OR: odds ratio.

## Discussion

### Principal Findings and Significance

By using a novel, comprehensive area-based SDoH-index called the DII, this investigation showcased significant associations between increasing levels of digital inequity with nasal-paranasal sinus cancer outcomes in care and prognosis. Overall, NPSC surveillance and treatment receipt decreased with increasing levels of digital inequity. To our knowledge, this is the first and largest study to apply a national index using prior-validated multivariate models of evaluating digital inequity associations with NPSC care and prognosis while accounting for nondigital, traditional social determinants. In doing so, this study found significant, detrimental trends of surveillance and treatment receipt with increasing digital inequity.

### Comparison to Prior Work

With nasal cavity cancers having high recurrence rates and rapid progression of such recurrences [3,14], understanding how surveillance is affected by factors of digital inequity becomes of utmost importance. As we showcased significant decreases in the length of follow-up associated with increasing DII, our study highlights how present technological disparities could exacerbate lacking surveillance for cancer recurrence among socially vulnerable populations. Moreover, beyond the window of high risk of primary NPSC recurrence within 2 years, understanding mechanisms of unequal follow-up, such as the contributions of digital inequity, beyond this length of time points to the observed 33% chance that patients with NPSC will develop a second primary head and neck cancer [14]. Further studies also showed that, although advances in imaging and surgery may help to decrease the rates of recurrence and secondary malignancy occurrence, diagnosis at an earlier stage, whether primary or recurrent disease, was the largest contributor to prognosis [15]. In turn, for a disease with such a high rate of recurrence and increased risk of a second primary malignancy, understanding how digital inequities impact long-term and lifelong follow-up and surveillance is crucial.

Our findings of digital inequity associations with NPSC help contextualize prior investigations of technology use in contributing to earlier diagnosis of primary malignancies. For instance, a study in Italy evaluated the use of telemedicine in breast cancer and cardiovascular disease detection and reported that telemedicine screening allowed for significantly earlier detection [16]. Furthermore, another study evaluated the use of telemedicine and monitoring in head and neck cancers, finding that a recent telemedicine model by Beswick et al [17] allowed for quicker access to surgery and increased financial benefit among patients with head and neck cancer [18]. In addition, telemonitoring in patients with head and neck issues has been shown to detect early occurrence of health problems [18]. Earlier detection of disease using digital advancements may allow for the detection of cancers at earlier stages. As such, these prior investigations infer that early screening technologies that lead to superior head-neck cancer outcomes, such as NPSC, would depend on digital resource accessibility. These relationships suggest possible mechanisms of how digital resource inequity associations observed in this study could inversely contribute to worse outcomes by limiting this early step in cancer diagnosis and the eventual cause of treatment delay, which is especially relevant to rarer cancers with incidental symptoms such as NPSC. However, to fully characterize this possibility, future prospective investigations across multiple patients with NPSC demographics should observe individual-level circumstances related to their surrounding digital inequity factors that may pose a potential prognostic difference.

As the mainstay of NPSC treatment involves both surgical intervention and radiation therapy [14], our study showcases how worse digital access is associated with unequal receipt of these indicated modalities akin to prior traditional SDoH studies showcasing similar disparities. Specifically, prior studies have found significant differences among individuals with paranasal sinus cancer and differing SES. A cross-sectional analysis by Sharma et al [5] found that among those with regional/distant disease, those in the middle and lowest SES tertile were significantly less likely to receive multimodal therapy. Furthermore, a radiation therapy noncompliance study involving the head and neck among other cancers found that low SES was associated with radiation therapy noncompliance, defined as missing 2 or more scheduled radiation therapy appointments [19]. Given that our analyses account for both nondigital SDoH, such as SES, and digital factors, our observed associations of treatment disparities among patients with NPSC necessitate further exploration of how digital resource inequities can affect present-day health outcomes.

Despite the numerous geospatial, national indices assessing traditional social determinants, the absence of tools to assess technological and internet-access factors in a similar, multifactorial fashion necessitated the use of the DII. Aside from using a validated set of measures in its multivariate settings, the DII reached the quantitative veracity of these established indices, such as the Social Vulnerability Index or Area Deprivation Index, by sourcing from identical, publicly available databases, with those being the ACS/US Census and Federal Communications Commission. Our application of the DII through the differential weighing of these ranked scores to form composite measures of social determinant themes remains aligned with the statistical considerations used by prior work in large-data SDoH-contextualizations in otolaryngology, alongside the application of DII to gastrointestinal-aerodigestive cancer disparities [13,20]. In turn, the application of the DII in this study was well-warranted for assessing the lesser-investigated factors of digital inequity in a geospatial, multifactorial manner.

### Strengths and Limitations

This study used a comprehensive novel index to assess a wide variety of digital inequity determinants and the potential impacts they may have on NPSC surveillance and treatment plans. Additionally, our populace was large for such a rare malignancy and consisted of individuals across all regions in the United States.

The authors recognize that this study had several limitations. First, this study was localized to the United States. Second, our populace was majority male and White. Although this accurately represents the majority of patients with NPSC, future studies exploring the relationship between the chosen strata for this study and the impact of observed DII should be investigated further. Third, the validated set of variables used in the DII does not encompass the entirety of digital and nondigital factors that would be of interest and follow the convention of its predecessors. Last, although the chronology of patients and DII measures was aligned, further actions to conduct updated investigations for more recent time periods (given the changes in digital resource access) would be warranted.

### Conclusions

In conclusion, using the validated multivariate models of the DII, disparities in NPSC follow-up and treatment were significantly associated with national digital inequities while accounting for traditional SDoH factors such as SES, education, and disability. Using novel, large-data tools such as the DII presents the means of identifying vulnerable sociodemographics and modern-day factors to inspire discourse on how digital resources affect care delivery and access. In turn, these findings further facilitate investigation into how equitable NPSC care and outcomes play out in modern-day environments while highlighting key targets for policy makers and public health advocates to conduct prospective initiatives against.

## Supplementary material

10.2196/52627 Multimedia Appendix 1 Variables used for Digital Inequity Index development.

## Abbreviations

ACS: American Community Survey
DII: Digital Inequity Index
HIPAA: Health Insurance Portability and Accountability Act
*ICD-O-3*: *International Classification of Diseases for Oncology, Third Edition*
NCI: National Cancer Institute
NPSC: nasal and paranasal-sinus cancer
OR: odds ratio
SDoH: social determinants/drivers of health
SEER: Surveillance, Epidemiology, and End Results Program
SES: socioeconomic status
