# Supplementary material for: The Impact of Digital Inequities on Nasal and Paranasal-Sinus Cancer Disparities in the United States: A Cohort Study
Source: JMIR Cancer. 2025 Jul 15;11:e52627. doi: 10.2196/52627 (PMC12282939; doi:10.2196/52627)
Supplement: Multimedia Appendix 1 [file cancer-v11-e52627-s001.docx]

**Table S1. Variables used for DII-development.**

| **Infrastructure-Access** | **Sociodemographic** |
| --- | --- |
| Households without a desktop or laptop  **[Device Access]** | 25+ years-aged people without a high-school diploma |
| Without access to non-mobile broadband  **[Internet Availability]** | 25+ years-aged people without an associate’s degree or higher |
| Without access to Broadband – DSL  **[Internet Availability]** | 25+ years-aged people without a bachelor’s degree or higher |
| Without access to Broadband – Cable  **[Internet Availability]** | Below poverty level within the last 12 months |
| Without access to Broadband – Fiber  **[Internet Availability]** | Below 150% of the poverty level within the last 12 months |
| Without access to Broadband - Terrestrial Fixed WiFi  **[Internet Availability]** | Disability status pertaining to cognitive, ambulatory, or self-care difficulties |
| Without mobile or non-mobile internet subscription of any type  **[Internet Availability]** |  |
| Without an internet subscription of cable, fiber, or DSL  **[Internet Availability]** |  |
| Without a broadband subscription in households $20,000 or less  **[Income-Access]** |  |
| Without a broadband subscription in households making $20,000 - $74,999  **[Income-Access]** |  |
| Without a broadband subscription in households making $75,000 or more  **[Income-Access]** |  |
